# 3D Printed Microfluidic Probes

**DOI:** 10.1038/s41598-018-29304-x

**Published:** 2018-07-20

**Authors:** Ayoola Brimmo, Pierre-Alexandre Goyette, Roaa Alnemari, Thomas Gervais, Mohammad A. Qasaimeh

**Affiliations:** 1 Division of Engineering, New York University Abu Dhabi, Abu Dhabi, UAE; 2 Department of Mechanical and Aerospace Engineering, New York University, New York, USA; 3 Institut de génie biomédical, École Polytechnique de Montréal, Montréal, Canada; 4 Department of Engineering Physics, École Polytechnique de Montréal, Montréal, Canada; 5 Centre de recherche du Centre Hospitalier de l’Université de Montréal, Montréal, Canada

## Abstract

In this work, we fabricate microfluidic probes (MFPs) in a single step by stereolithographic 3D printing and benchmark their performance with standard MFPs fabricated via glass or silicon micromachining. Two research teams join forces to introduce two independent designs and fabrication protocols, using different equipment. Both strategies adopted are inexpensive and simple (they only require a stereolithography printer) and are highly customizable. Flow characterization is performed by reproducing previously published microfluidic dipolar and microfluidic quadrupolar reagent delivery profiles which are compared to the expected results from numerical simulations and scaling laws. Results show that, for most MFP applications, printer resolution artifacts have negligible impact on probe operation, reagent pattern formation, and cell staining results. Thus, any research group with a moderate resolution (≤100 µm) stereolithography printer will be able to fabricate the MFPs and use them for processing cells, or generating microfluidic concentration gradients. MFP fabrication involved glass and/or silicon micromachining, or polymer micromolding, in every previously published article on the topic. We therefore believe that 3D printed MFPs is poised to democratize this technology. We contribute to initiate this trend by making our CAD files available for the readers to test our “print & probe” approach using their own stereolithographic 3D printers.

## Introduction

**Figure 1:**
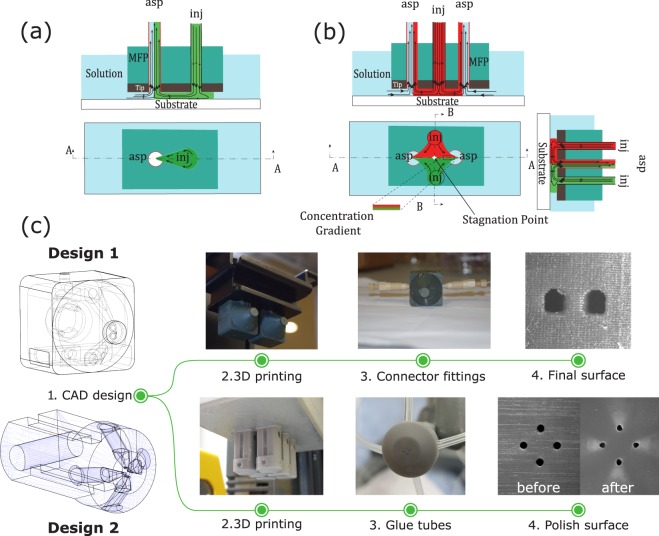
3D Printed MFPs. (**a**) MD configuration of the MFP and its operating principles. (**b**) MQ configuration of the MFP and its operating principles. (**c**) 3D printing fabrication steps for both designs of the MFP.

The microfluidic probe (MFP) is a non-contact microfluidic system that combines the concepts of hydrodynamic flow confinement (HFC)^1^ and scanning probes^2^ to yield a dynamic microfluidic device that eliminates the need to perform analyses within closed conduits. It operates under the well-known Hele-Shaw cell approximation, whereby a quasi-2D stokes flow is generated between two parallel flat plates separated by an arbitrarily small gap, and has been previously demonstrated in the microfluidic dipole (MD) and microfluidic quadrupole (MQ) configurations (see Fig. 1a,b). The technology, developed about a decade ago, is now well established, and has since then been iteratively developed and used by several groups mostly to perform open surface reagent patterning operations. Examples of the MFP’s applicatons include patterning protein arrays on flat surfaces^3^, mammalian cell stimulation and manipulations^3–5^, localized perfusion of tissue slices^6,7^, and generating floating concentration gradients^8^. Recently, the MD configuration of the MFP was proposed as a tissue lithography tool^9^, where it allowed for retrospective studies on formalin-fixed paraffin-embedded tissue sections. On the other hand, the MQ configuration was lately applied as a tool for advanced cell chemotaxis studies^10^, where it allowed for studying cellular dynamics during migration in response to moving concentration gradients.

The main advantage of the MFP is that it overcomes major limitations of conventional channel-based microfluidics^11,12^, such as high shear stress and tiny patterning areas, while allowing localized delivery of reagents for biological applications^13,14^. Another major advantage of the MFP is its ability to pattern large, open planar surfaces using conventional lab equipment (such as cells on Petri dishes)^15^.

However, the potential of the MFP is still largely untapped in the life sciences due to several barriers^16,17^. One of this barrier is the fact that MFPs cannot be easily produced on demand due to their typically complex fabrication procedures. For example, the technology was originally designed with a silicon tip and Polydimethylsiloxane (PDMS) chip-to-world connector, which requires bulk micromachining of the silicon chip, fabrication of the PDMS block using soft lithography or micro-molding techniques, alignment and assembly of the different layers, and separate machining of the probe holder^3^. An attempt to standardize this fabrication procedure introduced the glass-silicon hybrid vertical MFP (vMFP) concept, with a gasket-integrated probe holder for a more compact world-to-chip interface^4^. This technique has been demonstrated to be very effective in fabricating MFPs with all fluidic apertures placed along a straight line, and is currently one of the most commonly adopted. However, its procedure requires costly microfabrication facilities, implies wafer bonding steps, is difficult to implement for arbitrary aperture arrangements, and results in long prototyping cycles. The 3D printing technology, which is already positively disrupting the development cycles of conventional microfluidic devices^18,19^, is poised to overcome these challenges. Not only does it afford a relatively seamless connectivity of several parts, it also offers a straightforward, simple, rapid, inexpensive, and yet robust methodology to manufacture these devices on demand (Fig. 1c). More specifically, 3D printing of MFPs is highly customizable, and allows limitless designs and probe configurations. As opposed to the vMFP technology, any number of apertures can be printed in a single step without imposing constrains on aperture positions or arrangments.

In this work, we perform the first demonstration of 3D printed MFP operation by using two independently designed chips. The side-by-side comparison approach adopted throughout this paper seeks to emphasize the flexibility and universality of 3D printed MFPs. Throughout, we highlight the benefits of the method as well as the potential complications that may arise during the process, and present maneuvering strategies of such complications. By presenting two different designs that used different approaches and focused on different aspects, the objective is to show the different design and fabrication possibilities, and hint at the broad applicability using virtually any moderate to high resolution printers. To conclude, we validate the MFP’s effectiveness by performing a standard staining of living adherent cells in Petri dishes. Overall, this article aims to give guidelines that might assist other research groups in 3D printing probes suitable for their own application.

## Results

### Design of 3D MFPs

**Figure 2:**
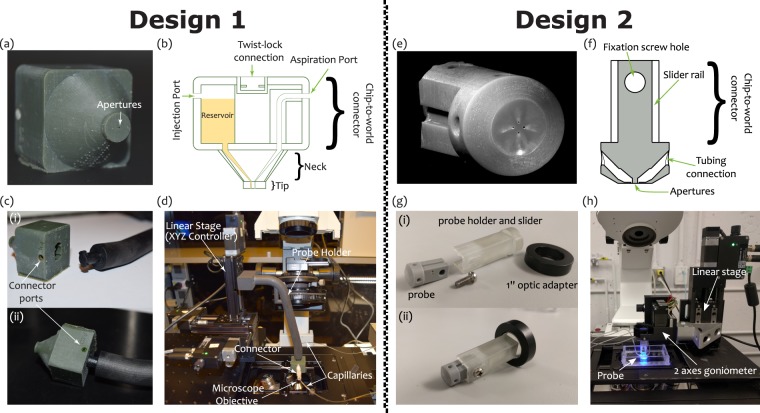
MFP Design 1 (**a**–**d**) and Design 2 (**e**–**h**) setups. Design 1: (**a**) MD MFP (**b**) Cross section schematic of the MFP. (**c**) Twist lock fastening mechanism for connecting the MFP to the probe holder: (i) before and (ii) after fastening. (**d**) Experimental setup mounted on an inverted microscope. Design 2: (**e**) MQ MFP. (**f**) Cross section schematic of the MFP (**g**) MFP holder showing the probe holder slider and the 1″ optic adapter: (i) before and (ii) after assembly. (**h**) Experimental setup mounted on an inverted microscope.

Two independent MFP designs, printers and setups were used in fabricating the MD and MQ MFPs. Both designs have cylindrical tips and conical necks, while differing in their world-to-chip interface. The first MFP (arbitrarily called Design 1), as shown in Fig. 2a, was fabricated using a commercial 82 µm resolution stereolithography printer (MicroPlus XL, EnvisionTEC GmbH, Gladbeck, Germany). This embodiment incorporates inbuilt reservoirs to store injection liquids, a “twist-lock” fastening mechanism to connect the MFP to a probe-holder fabricated in-house (also using a 3D printer), and ports that fit with commercially available tubing connectors (Fig. 2b). The inbuilt reservoir is located in the hollow square section, and serves as a more compact controlled storage environment for expensive samples. The twist-lock mechanism is inspired by the GU24 lamp fitting^20^. To connect the MFP using this mechanism, the two pins on the probe holder are inserted into the corresponding holes in the MFP and locked into place by twisting (Fig. 2c). The probe holder is then mounted on the probe station, Fig. 2d. Ports for tubing connectors were integrated with the MFP to minimize dead volume and provide leak-free system without using glue or gaskets. The channels connecting the tubing ports to the apertures are tapered through the conical and cylindrical sections – with their diameter gradually reducing to the final aperture size at the tip.

The second MFP (arbitrarily called Design 2) aims at maximum design simplicity with a commercial 27 µm resolution stereolithography printer (Asiga Pico Plus 27, Alexandria, Australia). There is no built-in reservoir in the MFP, and the fluidic connections are simply made by plugging commonly available 1/32” tubes into the probe and gluing them in place. The result minimizes probe size within the limits imposed by the printer (Fig. 2e,f). Special care was taken to obtain a perfectly flat tip and a symmetric aperture in this design. Thus, MFPs were printed vertically (which reduce the aperture asymmetry problem), and the MFP’s tips were polished using fiber optics lapping sheets. Two resins have been tested for this design. The PlasCLEAR V2 (Asiga) resin was proven to achieve the highest resolution, yielding MFP apertures as small as 150 µm. However, this resin is transparent and the MFP’s channels are visible when imaging fluids flow under it (Fig. S1a), which increases background signal and, reciprocally, decreases measurement sensitivity. A matte black ink coating and a clear coat applied on the MFP’s tips was found to solve this problem (Fig. S1b). However, since the coating increases the complexity of the fabrication process and might not be compatible with all applications, MFPs were also fabricated using gray resin (PlasGRAY V2, Asiga). With this resin, the resolution is slightly lower as such, the smallest repeatable apertures with this resin are 180 µm. The gray resin is not perfectly opaque (Fig. S1c), so images taken with these MFPs require a background subtraction (Fig. S1d).

In this design, the MFP and its adapter form a slider (Fig. 2g). This allows for an easy calibration of the MFP-to-substrate gap. The MFP is simply slid to the sample surface (so the gap is effectively zero) and then locked in place by a screw. The MFP can then be elevated by the required gap. The MFP adapter is compatible with a standard 1″ optic mount, which has been found to be a convenient standard to connect the MFPs on positioning systems made with opto-mechanical parts.

Akin to classical MFPs, these 3D printed MFPs are connected to micro-positioners and goniometers, that can be controlled in one or more axes, to control the MFP-to-substrate gap and angle of inclination respectively (Fig. 2d,h). The MFP stations used are compatible with almost any inverted microscope.

### Microfluidic Dipoles and Quadrupoles

The MD and MQ are produced using the 3D printed MFPs from both designs. The profiles were investigated as functions of MFP’s aperture Spacing to Diameter ratio (S/D), aspiration to injection flow rates ratio ($\alpha ={Q}_{asp}/{Q}_{inj}\,)$, and aspiration flow rate.

**Figure 3:**
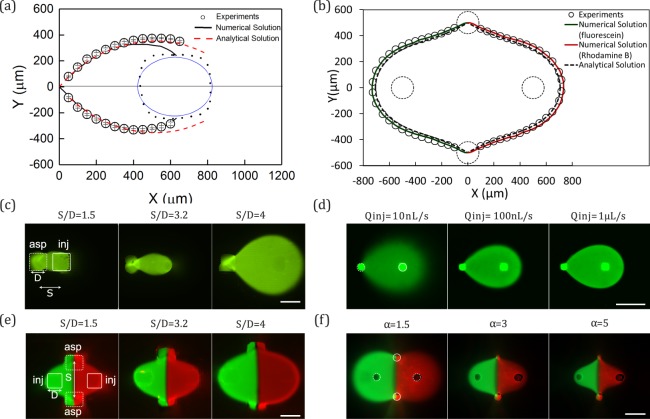
Characterization of MD and MQ produced by the 3D printed MFPs. (**a**) Comparison between the experimentally measured, numerical simulation, and analytically calculated flow profiles of the MD using Design 1 MFP. D = 400 µm, α = 3. (**b**) Comparison between the experimentally measured, the numerical simulation, and analytical flow profiles of the MQ using the Design 2 MFP with fluorescein (left) and rhodamine B (right). D = 180 µm, α = 3. (**c**) MD profiles produced using the Design 1 MFP as a function of aperture spacing to diameter ratio (S/D). Q_*inj*_ = 100 nL/s, α = 5. (**d**) MD profiles of the Design 2 MFP (clear resin MFP coated with black ink) as a function Q_*inj*_. S/D = 4.8, α = 3. (**e**) MQ profiles produced using Design 1 MFP as a function of S/D. Q_*inj*_ = 100 nL/s, α = 3. (**f**) MQ profiles produced using Design 2 MFP (gray resin)as a function of *α*. Q_*inj*_ = 100 nL/s, S/D = 5.5. All scale bars are 500 µm.

Experimental measurements of the MD profiles used for the theoretical comparison were repeated at least 3 times with different 3D printed MFPs to test for reproducibility. With Design 1, while it was evident that the printed apertures contained some artifacts perturbing the circular shapes inputted in the CAD drawing (see blue dotted profile in Fig. 3a), the numerical model apertures were kept circular to visualize the resulting errors due to theses artifacts. Design 2 MQ apertures were more circular (Fig. 3b) but its material was slightly transparent, so a background subtraction was required (see Fig. S2 for complete process).

When using the MFP for surface patterning, the dimensions of the HFC area dictate the shape and size of the “brush stroke”, hence precise control over the dimensions of the HFC zone is valuable as a user-controlled parameter^21^. In the present work, the experimentally measured MD profiles were observed to be reproducible within a reasonable limit (see error bars in Fig. 3a). In addition, the measured MD profiles match the trend of the profiles calculated using mathematical models (analytical and numerical), and majority of the points lie within a 20% error margin. However, as shown in Fig. 3a, slight asymmetry can be observed in the MD measured profile when its correlation with the calculated profiles is closely examined–especially at regions closer to the injection aperture. This can be attributed to the error contribution of the printed artifacts and misalignment of the MFP in parallel with the bottom substrate. Comparisons between modelled and experimentally measured profiles using other values of *α* depict the same trend. However, based on our observation, as *α* increases, the analytical model increasingly underestimates the profile size, in comparison to the experimental and numerical calculated profiles (see Fig. S3). This can be attributed to the point source approximation utilized in formulating these models^21^.

Comparison between numerical simulations and experiments for the MQ profiles (Fig. 3b) shows the expected symmetrical HFC area, with, also as expected, a slightly more diffuse rhodamine interface due to a larger diffusion coefficient (3.4 × 10^−9^ m^2^/s for rhodamine B and 5 × 10^−10^ m^2^/s for fluorescein^22^). Close inspection reveals that the location of the stagnation point was matched with a relative error of approximately 2%. This might be due to experimental inaccuracies such as an imperfect tip surface or small angle between the MFP and the bottom substrate. The analytical model also underestimates the HFC size by approximately 25 µm on all borders. This relatively small error (<5%) is explainable by the fact that the analytical solution considers point source approximation (and thus a S/D ≫ 1). For a similar S/D ratio than the one used in this experiment (S/D = 5.5), error of the same magnitude have been previously reported^21^. Also, the analytical model doesn’t consider the diffusion at the HFC interfaces. However, diffusion affects our experimental measurement and the threshold we used to determine the experimental HFC envelope might slightly overestimate it.

As expected, for an MD with a fixed *α* of 5, an increasing HFC footprint was obtained when S/D is varied from 1.5 to 4 (Fig. 3c). This trend is consistent when *α* is varied from 3 to 10 (Fig. S4). On the other hand, the HFC area is independent of the absolute values of the injection and aspiration flow rates – the difference in the boundary sharpness is due to diffusion broadening^8^. This trend is demonstrated when injection flow rates are varied from 10 nL/s to 1 µL/s, for a fixed *α* of 3 (Fig. 3d).

In a similar fashion to the MD, as S/D increases and *α* reduces, the HFC area of the MQ is expected to increase^3^, and these trends are also observed in our experiments as shown in Fig. 3e,f, respectively. However, the expected concentration gradient at the interface of the injected fluids is not visible in either of these figures due to the quenching of the rhodamine B by fluorescein^23^.

### Selective Staining of Live Adherent Cells

**Figure 4:**
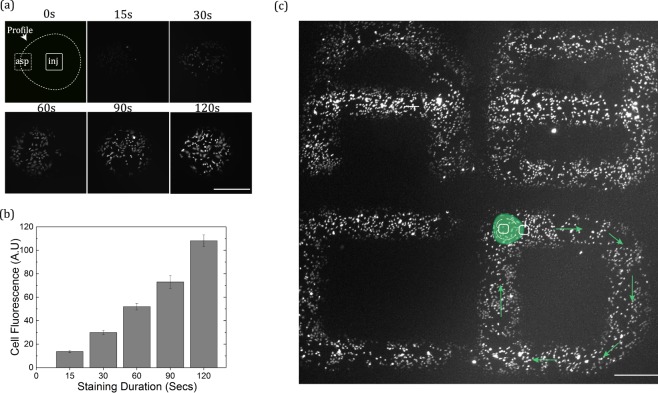
Selective staining of live adherent HeLa cells cultured in Petri dish. (**a**) Cell fluorescence as a function of staining duration. The value of α for all patterns are 2. (**b**) Quantification of the cell fluorescence intensity as a function of staining duration. Error bars indicate standard error of intensity measurements from 5 random cells. (**c**) Calligraphic patterns drawn at average scanning speed of 30 µm/s. Illustrations on letter “D” shows the programed path of the probe during the scanning movement. In letters A and B, the MFP made two scans over the horizontal lines (see Fig. S5), hence, their relatively higher staining intensity. All scale bars are 500 µm. Injection flow rates in (**a**) and (**c**) is 60 nL/s and MFP-substrate gap is 60 µm.

In order to test the practicability and biocompatibility of the 3D printed MFPs, a cell staining test similar to the demonstrations with the Si-based MFP^4^ was performed. To achieve this, HeLa cells were grown inside cell culture media with a confluence of about 40%, and the MFP was used to target a selected sub-population (~100 cell) of the culture with a solution of CellTracker® Green fluorescence dye (10 μM concentration), using a MFP-substrate gap of 60 µm. As expected, an increase in staining intensity is observed as the exposure duration of the cells to the HFC (of the CellTracker solution) is increased from 15–120 s (see Fig. 4a,b).

The developed MFP was also capable of staining cells while operating in the scanning mode (see Fig. 4c), with a residence time of a few seconds required over each cell cluster. This demonstration proves the capability of the device to selectively deliver reagents to local areas of sub-population of cells in a dynamic way or, potentially, to tissue slices, without disrupting their integrity or growth. Observations 24 h post experiment showed that the staining operation using these MFPs does not affect cell viability – as deduced from the observed (93 ± 3)% survival rate (mean ± standard deviation, 3 separate experiments), and the 39% cell proliferation rate that matches with control experiments (see Fig. S6). The path traveled and scanning speed of the MFP during the staining operation was pre-programmed according to Fig. S5. SI Video 1 and SI video 2 show the staining operation for average scanning speeds of 30 µm/s and 60 µm/s respectively. It took about 20 min and 40 min to complete patterns with the 30 µm/s and 60 µm/s speeds respectively.

## Discussion

This work demonstrates the applicability of stereolithography as a rapid, inexpensive and robust technique for fabricating MFPs. Fabricating MFPs using 3D printing is straightforward as it requires no alignment, bonding or etching procedures, which are generally the most challenging steps in fabricating Si, glass, and PDMS-based MFPs. They are fast to fabricate – all steps can be made in less than a day. They only require a few milliliters of resin for laboratories or companies that have access to a stereolithography printer (which are becoming increasingly popular), are relatively inexpensive via 3D mail-order printing services, and are potentially as reusable as Si-based MFPs. This approach allowed for the integration of new components like inbuilt reservoirs, convenient MFP probe holders, and using commercially available tubing connectors for a straight forward connectivity and minimization of dead volume–that would have been otherwise complicated to achieve with conventional MFP microfabrication techniques. Moreover, this work demonstrated how 3D printing brings scalability and flexibility to the MFP in terms of producing different designs, different numbers of apertures, different aperture configurations, and different fluidic connectors. Using two independent MFP designs, proof-of-concepts were presented in the form of developing MDs, MQs, and selective labeling of adherent live cells within their culture medium in a “writing” mode. The MD and MQ profiles produced by the 3D printed MFPs closely matched numerical and analytical calculations – the same way micromachined MFPs do – thus confirming similarities in their accuracies. Furthermore, cell staining applications were demonstrated, which validate the applicability of the 3D printed MFPs in performing bioassays.

The use of affordable 3D printing in this field, however, comes with some drawbacks: (1) the current resolution of the affordable 3D printers limits the minimum aperture sizes of MFPs. However, this didn’t affect the capability of presented MFPs to target cluster of tens of cells and generate concentration gradients at resolutions similar to the original MQ^8,10^. (2) The printed MFPs contained some unavoidable channel artifacts – one design more than the other – that had minor effect on the accuracy of the produced profiles. However, with 3D printing technology currently undergoing intense development to bring down costs and increase resolution, these limits are expected to be continuously pushed back as new generations of 3D printers come to the market. In the case an MFP is required for an application that requires a single cell resolution, the same technology discussed here can be adapted to more advanced 3D printers capable of printing 3D nanostructures, such as those involving direct laser writing and two-photon polymerization technologies^24,25^. (3) Biocompatibility of the polymers and resins used in SLA is still a concern for applications where cells are directly cultured on the device^26^. However, in the case of MFPs, cells are cultured in Petri dishes, or microtiter plates, and are never in contact with the 3D printed device, which limit the biocompatibility problem. Furthermore, research on new post-processing and sterilization techniques, and on biocompatible resin formulation, have been demonstrated to reduce protein adsorption and improve long-term cell viability after experiments with 3D printed materials^27–29^.

The HFC concept behind the MD and MQ have numerous potential applications, which could become even more accessible to the research community with this new fabrication strategy, lessons learned from our experiments, and the presented proof of its practical applicability. By demonstrating the simplicity and customizability of 3D printed MFPs, we believe that the technology will help spread the concept outside microfabrication laboratories and make its potential and versatility as an open reagent delivery tool directly available to life-science laboratories. To support this assertion, all CAD and stl files used to print the above designs are made available (see Supplementary Material) for any stereolithographic 3D printer owner to test.

## Materials and Methods

### 3D printing of MFPs

Design 1**:** Designs were performed using a commercial CAD software (SolidWorks) and the MFPs were printed using a commercially available stereolithographic 3D printer (MicroPlus XL, EnvisionTEC GmbH, Gladbeck, Germany). The printer has a labeled XY resolution of 82 µm, and Z resolution of 25 µm but our parametric study with this specific MFP design showed that the minimum diameter resolution for repeatable 3D channels is 250 µm using a polymeric resin (HTM 140 resin, EnvisionTEC GmbH, Gladbeck, Germany). After printing, the chip was washed with ethanol, placed in a UV flood light curing system for 2 min and then washed again with DI water. The MFP holder was separately 3D printed (Dimensions SST 1200es 3D Printer, Stratasys, Minnesota, US).

Design 2: Designs were made using Catia v5 (Dassault Système, Vélizy-Villacoublay, France) while abiding by two rules to minimize the risk of aperture clogging: 1) the length of apertures was kept the smallest possible (max 3 print layer long, 150 µm) and 2) aperture size was kept over 6 printer’s pixel size (160 µm). The MFPs were printed using an Asiga freeform Pico with a resolution of 27 µm (Asiga, Alexandria, Australia). The resins used were PlasCLEAR V2 and PlasGRAY V2 (Asiga). MFPs were briefly soaked in an isopropanol bath to remove uncured resin. To thoroughly clean the microchannels, the MFPs were then put in an isopropanol bath in a sonicator (Branson, Danburry, USA) for 20 minutes. Thereafter, MFPs were post-cured with UV light for 15 minutes to fully harden (Pico flash, Asiga). MFPs were designed to fit widely available 1/16″ O.D. tubing. Tygon tubes (Cole Parmer, Vernon Hills, USA) were plugged in the MFP and glued with cyanoacrylate glue. To insure no glue would enter inside the MFP apertures and tubing, each tube was filled with water during this step. MFP tips were manually polished using fiber optic lapping sheets (Thorlabs, Newton, USA). Sheet with 5 µm, 3 µm, 1 µm and 0.3 µm grits were used consecutively. Water was injected with the MFP’s apertures after each polishing steps to make sure no polishing residues would clog the apertures. MFPs made with clear resin were coated with black ink (black Indian ink, Winsor & Newton, London, UK) using an airbrush. A layer of aerosol clear coat (Protective clear coat finish, Dupli-color, Cleveland, USA) was then sprayed on the tip.

### Experimental Characterizations

To characterize the MD and MQ, the MFP was positioned above a Petri dish or a microscope slide with custom walls (depending on the design) located on the stage of an inverted microscope. Upon aligning the MFP with the objective of the microscope, the Z stage (X-LRM, Zaber, Vancouver, Canada) is used to slowly approach the substrate. When the MFP contacts the substrate, it is then lifted by the required gap-height to produce the Hele-Shaw two parallel plates configuration. The Design 1 MFPs were visualized using the Epi-fluorescence inverted ECLIPSE Ti microscope (NIKON,Tokyo,Japan), and images were captured using the DS-Qi2 camera (NIKON). Design 2 MFPs were visualized and imaged using the epi-fluorescence inverted microscope Axio Observer.Z1 (Zeiss, Oberkochen, Germany) and the LaVision imager sCMOS camera (LaVision, Göttingen, Germany). MD images were taken by injecting a solution of dissolved green fluorescein sodium salt (C_20_H_10_Na_2_O_5_) (Sigma Aldrich, Saint-Louis, USA). For the MQ, a solution of Rhodamine B (C_28_H_31_ClN_2_O_3_) (Sigma Aldrich) was injected through the second injection aperture. Injection and aspiration were driven using the MFCS®-EZ microfluidic pressure pump (Fluigent, Kremlin-Bicêtre, France) or by the neMESYS high precision syringe pump (CETONI, Korbußen, Germany) depending on the design used. Images of the profiles created by fluorescein and rhodamine B solutions were captured using their respective microscope’s fluorescent filter cubes, and then combined using Image J (Design 1) (National Institutes of Health Maryland) and MATLAB (Design 2). For Design 2 MQ, a background image was subtracted on both fluorescein and Rhodamine B channels before they were merged in a single image. The background used corresponds to an image of the MFP with injection or aspiration turned off. To deduct the diffusion region from the overall profile, we used the “Minimum Threshold” function in ImageJ, which was derived from the thresholding technique adapted in reference^30^. Profiles were then traced out using a plot digitizer (GNU Library). For the dipole characterization, the experiment was repeated with at least 3 different MFPs per profile to test reproducibility. The dimensions for each interval was averaged to produce the final plot with standard error estimates used as the error bars.

### Numerical Modeling

The 3D numerical models of the MD and MQ were developed using finite element simulations (COMSOL Multiphysics® v.5.2. COMSOL AB, Stockholm, Sweden). The solutions were obtained using a direct and stationary solver to compute the Navier-Stokes equation (laminar and no-slip wall conditions) coupled with the convection-diffusion equations. MFPs were modeled as circular surfaces with inlets and outlets that correspond to the apertures. Dimensions used were those of the designed MFPs. Water was considered incompressible with a density of 998.2 kg/m^3^ and a dynamic viscosity of 0.001 N·s/m^2^. Diffusion coefficients used were 0.5 × 10^−9^ m^2^/s for fluorescein and 3.4 × 10^−9^ m^2^/s for rhodamine B, which correspond to the diffusion coefficients of these fluorophores in water^22^. The boundary conditions at the flow domain’s perimeter sides were set as a pressure outlet with atmospheric conditions, and no slip condition was selected for the MFP tip and the substrate surface. The experimental values were used for injection and aspiration volume flow rates. For the convection-diffusion equations, a zero-inward flux was set at the perimeter of the flow domain. The numerical solution of the MQ was computed for fluorescein and rhodamine separately and then superposed. The same methodology was used to deduct the diffusion region from the experimentally measured profile (thresholding and profile tracing) was used on the numerical simulation result.

### Analytical Modeling

Exact solutions for the MD HFC profiles under the point source aperture approximation are defined by the streamline obeying the following equation^21^:

$$-\arctan (\frac{{\check{x}}-1}{{\check{y}}})+{\rm{\alpha }}\,\arctan (\frac{{\check{x}}+1}{{\check{y}}})=\frac{\pi (\alpha -1)}{2}$$

where ${\check{x}}$ and ${\check{y}}$ are defined as 2*x*/*S* and 2*y*/*S* respectively (see schematic description of *α*, *x*, *y and S* in Fig. 3). This result was obtained by deducing the stream function for MD and then finding the equation for the streamline passing by the stagnation point^21^. Using the same method, the MQ’s HFC area was determined using the stream function and the stagnation point for the MQ^8^. This yields the equation:

$$-\arctan (\frac{x-\frac{S}{2}}{y})-\arctan (\frac{x+\frac{S}{2}}{y})-{\rm{\alpha }}\,\arctan (\frac{x}{\frac{S}{2}-y})+{\rm{\alpha }}\,\arctan (\frac{x}{\frac{S}{2}+y})=-\,\pi $$

These equations consider only the streamline, and thus, neglects diffusion. The variables in the equation cannot be separated hence, for each value of ${\check{x}}$, values of ${\check{y}}$ were calculated using WolframAlpha (Design 1) and MATLAB (Design 2).

### Selective cell labeling

HeLa cells (ATCC, VA) were cultured in sterile Petri Dishes (Thermo Scientific) in Dulbecco’s Modified Eagle Medium (DMEM) supplemented with 10% FBS, 100 U/mL penicillin, 100 μg/mL streptomycin and 0.2 mM L-glutamine (Invitrogen, MA) at 37 °C and 5% CO_2_. Dishes were left in the incubator until the time of the experiment. CellTracker® Green BODIPY (Thermo Fisher Scientific) diluted in PBS buffer with a final concentration of 10 μM/mL. The solution was then mixed with fluorescein sodium salt (final concentration of 1 mg/mL) for visualizing the HFC using the microscope. The solution was then loaded into the MFP’s reservoir – by suction through the aperture of the MFP – and the MFP was positioned above the cell culture with a MFP-substrate gap of 60 µm. Monitoring of the cells’ exposure time was performed using a stopwatch. The calligraphic labeling was performed by programming (C#) the XY stage movements in the Zaber scripting environment. The spacing between each letter was achieved by rapid movement of the stages (~29 mm/s), from the end of one letter to the beginning of the other letter (See Supplementary Video 1, and SI 2).

MFPs used with cells were sterilized by soaking in 99% ethanol for 24 h. Washing with DI water, and curing, were then repeated and followed by soaking in DMEM for 12 h. Before preparing cell dishes used for the cell viability experiment, marks were randomly made at the bottom of the culture plates to ensure traceability of the target region. Cell viability was estimated by applying NucGreen™ Dead Cell Indicator (2 drops/mL of cell medium) to the cell culture dish already exposed to the HFC from the MFP. As control, a cell dish prepared with the same cell confluence as the processed dish, was kept in the incubator for the duration of the experiment and also processed with the NucGreen™ Dead Cell Indicator (Thermo Scientific) after 24 h. Counting of cells to evaluate death rate (number of dead cells after 24 h/total number of cells before experiment), was done manually using the ImageJ “Multi-Point” feature.

## Electronic supplementary material

Supplementary Figures and Materials

Automated cell labeling 1

Automated cell labeling2
