# Supplementary material for: 3D Printed Microfluidic Probes
Source: Sci Rep. 2018 Jul 20;8:10995. doi: 10.1038/s41598-018-29304-x (PMC6054653; doi:10.1038/s41598-018-29304-x)
Supplement: Supplementary file 1 — Supplementary Figures and Materials [file 41598_2018_29304_MOESM1_ESM.pdf]

Supplementary Figures

3D-Printed Microfluidic Probes

Ayoola Brimmo,[1,2*] Pierre-Alexandre Goyette,[3*] Roaa Alnemari,[1] Thomas Gervais,[3†] Mohammad A. Qasaimeh [1,2†]

1. *Division of Engineering, New York University Abu Dhabi*
2. *Department of Mechanical and Aerospace Engineering, New York University, New York*
3. *Institut de génie biomédical, École Polytechnique de Montréal, Montréal*
4. *Department of Engineering Physics, École Polytechnique de Montréal, Montréal*
5. *Centre de recherche du Centre Hospitalier de l'Université de Montréal, Montréal*

**Equal contribution*

† Correspondence should be addressed to:

thomas.gervais@polymtl.ca; mohammad.qasaimeh@nyu.edu

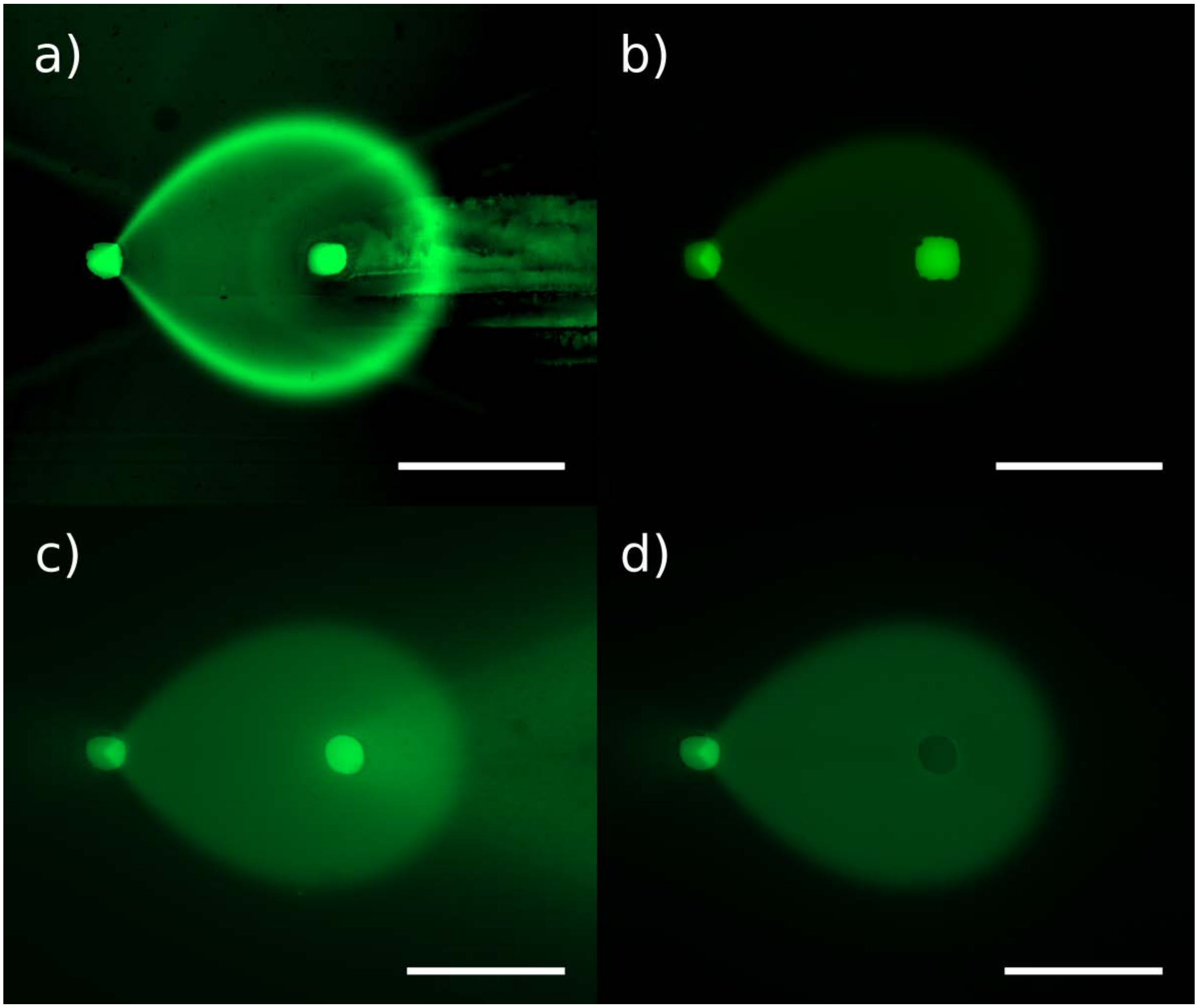

Fig. S1. Design2 strategies to reduce background noise. (a) Clear resin probe. (b) Clear resin probe with black ink and clear coat (c) Grey resin probe. (d) Grey resin probe with background subtraction. Injection flow rate = 100 nL/s, α=3, scale bar = 500μm

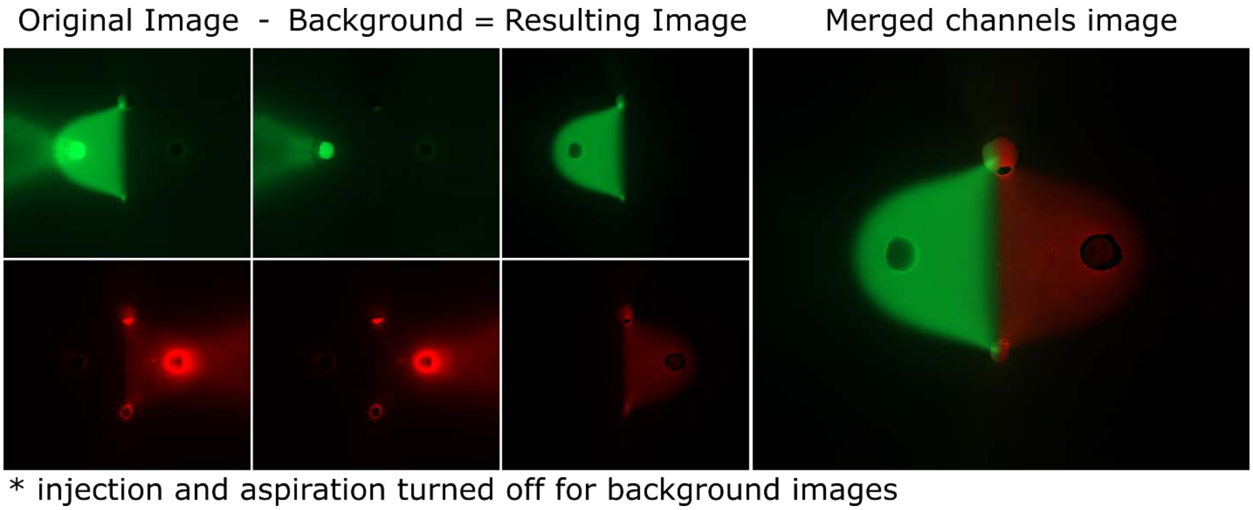

Fig. S2. Detailed background subtraction process for quadrupole. For the green and red channel, a background image of the probe with apertures turned off is subtracted to the working probe images. Green and red channels are then merged.

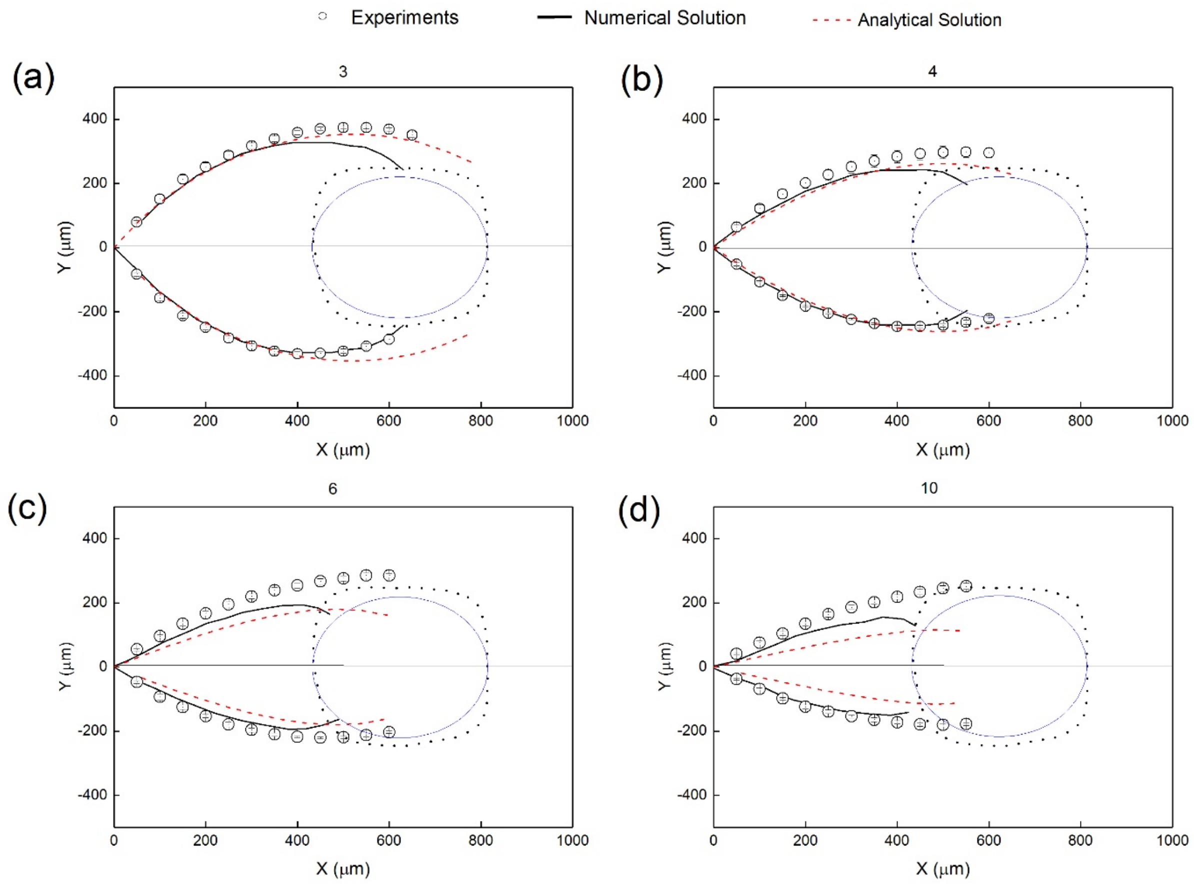

Fig. S3. Characterization of the MD profile with varying α. (a) α= 3. (b) α=4. (c) α=6. (d) α=10.

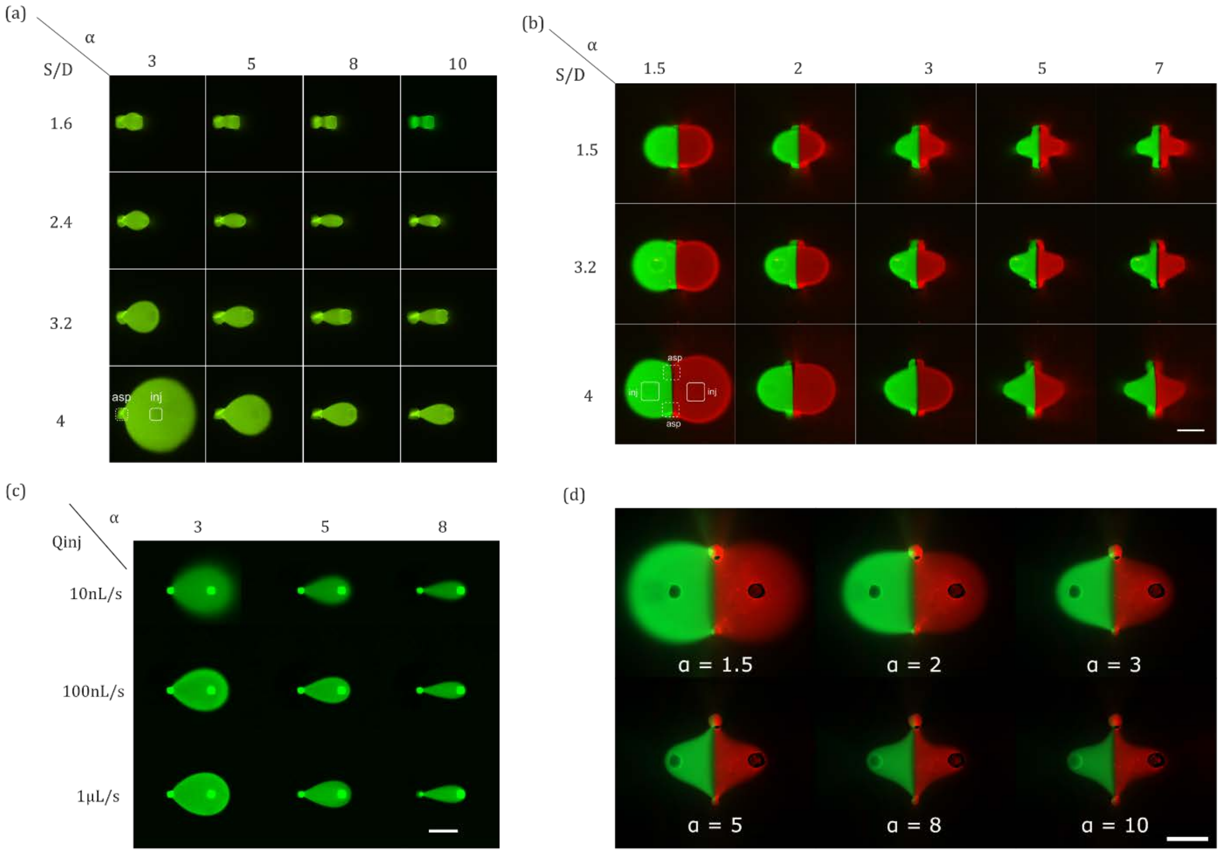

Fig. S4. Dipole and Quadrupole template profiles as a function of S/D, Q_{inj}, and α. (a) Dipole profiles produced by Design 1 MFPs as a function of S/D and α.(b) Quadrupole profiles made with Design 1 MFP as a function of S/D and α. (c) Dipole profiles produced by Design 2 MFPs as a function of Q_{inj}, and α. (d) Quadrupole profiles made with Design 2 MFP for different α. scale bar = 500μm

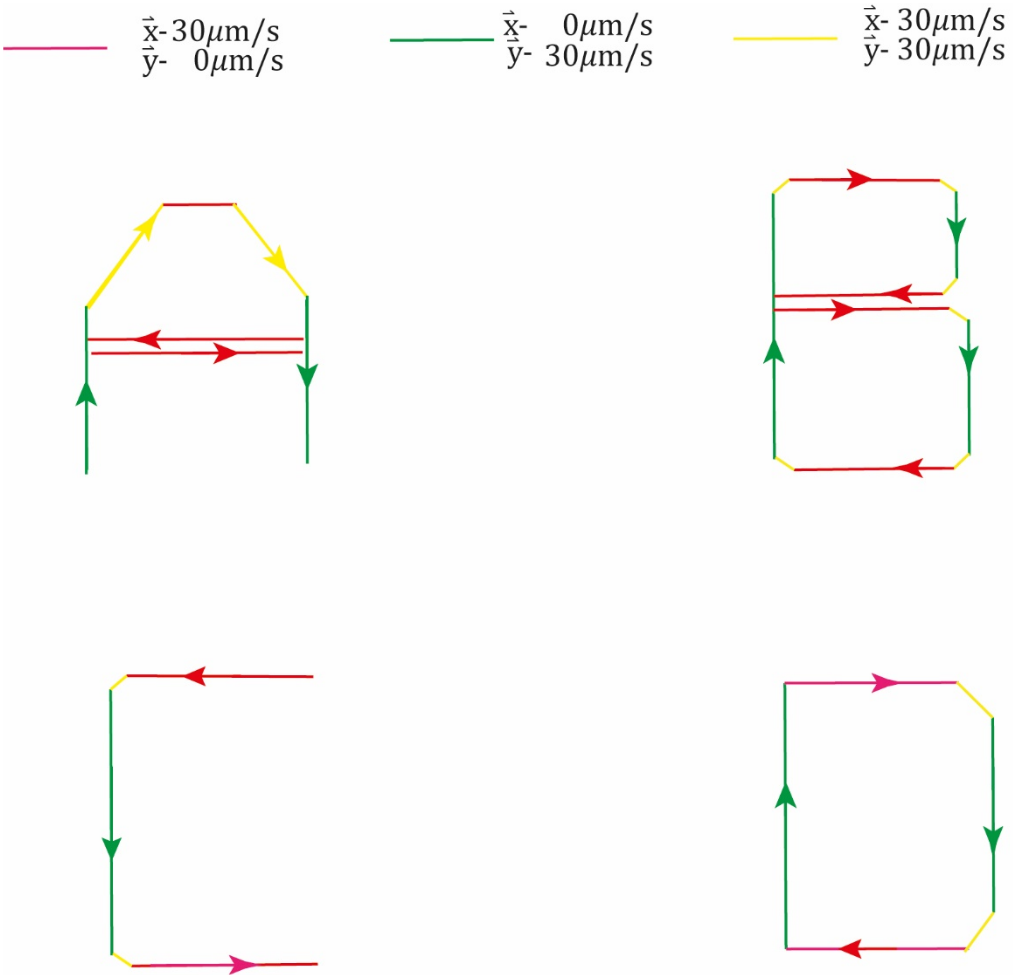

Fig. S5. Stage movement path for calligraphic cell labelling

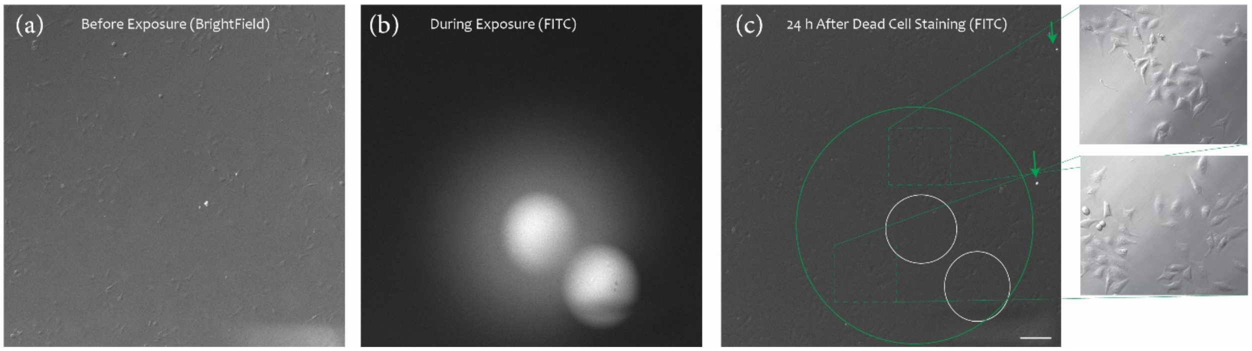

Fig. S6. Cell viability experiments. (a) Target cell region before exposure to HFC (bright field image). (b) HFC of green fluorescein sodium salt above targeted cells. (c) Targeted region with dead cell staining 24 h after experiment. Green arrows point at stained dead cells. Scale bar = 100μm

Supplementary Material

3D-Printed Microfluidic Probes

Ayoola Brimmo,[1,2*] Pierre-Alexandre Goyette,[3*] Roaa Alnemari,[1] Thomas Gervais,[3†] Mohammad A. Qasaimeh[1,2†]

1. *Division of Engineering, New York University Abu Dhabi*
2. *Department of Mechanical and Aerospace Engineering, New York University, New York*
3. *Institut de génie biomédical, École Polytechnique de Montréal, Montréal*
4. *Department of Engineering Physics, École Polytechnique de Montréal, Montréal*
5. *Centre de recherche du Centre Hospitalier de l'Université de Montréal, Montréal*

**Equal contribution*

† Correspondence should be addressed to:

thomas.gervais@polymtl.ca; mohammad.qasaimeh@nyu.edu

Location of STL Files

The STL files can be found at this link: https://wp.nyu.edu/amm_lab/download/

Script for Calligraphic Cell Labelling

```
// C# / JavaScript sample that demonstrates moving two devices simultaneously
// in binary mode.
#template(Simple)

// Get conversations for the X and Y devices (2 and 4).
var c2 = PortFacade.GetConversation(2);
var c4 = PortFacade.GetConversation(4);

// Start a topic to wait for the response
var topic = c2.StartTopic();

//Definitions
var Height = 44100;// Divide by 21 to get the real values in micrometers (convert steps to length)
var Space = 18900;

var Fast= 1000000;
var Normal= 1032; // Divide by 34.4 to get the real values in micrometers per second

//Set Speed to Normal
c4.Request(Command.SetTargetSpeed, Normal);
c2.Request(Command.SetTargetSpeed, Normal);

// Definitions of Heights
var Height75 = Height*0.75;
int height75 = (int) Height75;
```

```
var Height35 = Height*0.35;
int height35 = (int) Height35;
var Height30 = Height*0.30;
int height30 = (int) Height30;
var Height25 = Height*0.25;
int height25 = (int) Height25;
var Height50 = Height*0.50;
int height50 = (int) Height50;
//var S1 = H1*0.65;
//int s1 = (int) S1;


//**********************************************************/////
///------------------------------------------------------------////
// Start of Letter A
///------------------------------------------------------------////

// Vertical line with 75% total height
c2.Request(Command.MoveRelative, height75);

// Slanted line with 35% total height (Positive slope)
topic = c2.StartTopic();
c2.Device.Send(Command.MoveRelative, height25, topic.MessageId);
while ( ! topic.IsComplete)
{
		c4.Request(Command.MoveRelative, height25);
}


topic.Validate();

// Horizontal line with 50% total height
c4.Request(Command.MoveRelative, height50);

// Slanted line with 25% total height (negative slope)

topic = c2.StartTopic();
c2.Device.Send(Command.MoveRelative, -height25, topic.MessageId);
while ( ! topic.IsComplete)
{
		c4.Request(Command.MoveRelative, height25);
}


topic.Validate();

// Vertical line with 25% total height
c2.Request(Command.MoveRelative, -height25);

// Horizontal straight line to cross Letter A

c4.Request(Command.MoveRelative, -Height);
```

```
// Return cross A horizontal line
c4.Request(Command.MoveRelative,Height);

// Vertical line with 50% total height
c2.Request(Command.MoveRelative, -height50);
//Lower portion of Slanted A (45% of height)


//*******************************************************/////
///----------------------------------------------------------////
// Start of Spacing between A and B (Space Bar)
///----------------------------------------------------------////

// Increase Speed
c4.Request(Command.SetTargetSpeed, Fast);

// Move X stage to give space
c4.Request(Command.MoveRelative, Space);

//Reduce Speed back to normal
c4.Request(Command.SetTargetSpeed, Normal);



///----------------------------------------------------------////
// Start of Letter B
///----------------------------------------------------------////

//Definitions

// Top portion of B (20% of height)
var TopB = Height*0.2;
int topB = (int) TopB;

// Bottom Portion of B (40% of height)
var BottomB = Height*0.4;
int bottomB = (int) BottomB;


// Chamfer definitipn (10% of Height; 45 Degrees)
var Chamfer= Height*0.1;
int chamfer= (int) Chamfer;


// Straight vertical line (Y Direction)
        c2.Request(Command.MoveRelative, Height);



// Straight horizontal line (X Direction)
        c4.Request(Command.MoveRelative, Height);
```

```
// Chamfers
		topic = c2.StartTopic();
c2.Device.Send(Command.MoveRelative, -chamfer, topic.MessageId);
while ( ! topic.IsComplete)
{
		c4.Request(Command.MoveRelative, chamfer);
}

topic.Validate();

// Top B vertical line (Y direction)
		c2.Request(Command.MoveRelative, -topB);

// Chamfers
topic = c2.StartTopic();
c2.Device.Send(Command.MoveRelative, -chamfer, topic.MessageId);
while ( ! topic.IsComplete)
{
		c4.Request(Command.MoveRelative, -chamfer);
}

topic.Validate();


// Horizontal cross on Letter B (X Direction)
		c4.Request(Command.MoveRelative, -Height);
		c4.Request(Command.MoveRelative, Height);

// Chamfers
topic = c2.StartTopic();
c2.Device.Send(Command.MoveRelative, -chamfer, topic.MessageId);
while ( !topic.IsComplete)
{
		c4.Request(Command.MoveRelative, chamfer);
}

topic.Validate();

// Vertical line for bottom of B (Y direction)
		c2.Request(Command.MoveRelative, -bottomB);

// Chamfers
topic = c2.StartTopic();
c2.Device.Send(Command.MoveRelative, -chamfer, topic.MessageId);
while ( ! topic.IsComplete)
{
		c4.Request(Command.MoveRelative, -chamfer);
}

topic.Validate();

// Horizontal line to complete letter B (X direction)
		c4.Request(Command.MoveRelative, -Height);
```

```
topic.Validate();

//************************************************************//

///-----------------------------------------------------------////
// Returing Stage to begin letter C in line below (Enter Button)
///-----------------------------------------------------------////

//Definition
var re = Space + Height + Height;
int Re = (int) re;


//Speed up Movement (X-Direction)
c4.Request(Command.SetTargetSpeed, Fast);

// Move X stage in X direction back to Starting Position of C
c4.Request(Command.MoveRelative, -Space);

//Slow down Movement (X-Direction)
c4.Request(Command.SetTargetSpeed, Normal);

//Speed up Movement (Y-Direction)
c2.Request(Command.SetTargetSpeed, Fast);

// Give vertical spacing with Y stage
c2.Request(Command.MoveRelative, -Space);

//Slow down Movement (Y-Direction)
c2.Request(Command.SetTargetSpeed, Normal);


///-----------------------------------------------------------////



///-----------------------------------------------------------////
// Start of Letter C
///-----------------------------------------------------------////
/// Definitions
// Vertical C line (80% of height)
var VerticalC = Height*0.8;
int verticalC = (int) VerticalC;



// Chamfer definition (10% of Height; 45 Degrees)
var ChamferC= Height*0.1;
int chamferC= (int) ChamferC;
```

```
// Horizontal Line backwards (X direction)
c4.Request(Command.MoveRelative, -Height);

// Chamfers
            topic = c2.StartTopic();
c2.Device.Send(Command.MoveRelative, -chamferC, topic.MessageId);
while ( ! topic.IsComplete)
{
            c4.Request(Command.MoveRelative, -chamferC);
}

topic.Validate();

// Vertical line downwards (Y direction)
            c2.Request(Command.MoveRelative, -verticalC);


// Chamfers
            topic = c2.StartTopic();
c2.Device.Send(Command.MoveRelative, -chamferC, topic.MessageId);
while ( ! topic.IsComplete)
{
            c4.Request(Command.MoveRelative, chamferC);
}

topic.Validate();

// Horizontal Line Forward (X direction)
c4.Request(Command.MoveRelative, Height);


//***********************************************************//

///---------------------------------------------------------////
// Spacing between C and D (Space Bar)
///---------------------------------------------------------////

//Definition

//Speed Movement (X-Direction)
c4.Request(Command.SetTargetSpeed, Fast);
// Move X stage to give space
            c4.Request(Command.MoveRelative, Space);
            //Slow down Movement (X-Direction)
c4.Request(Command.SetTargetSpeed, Normal);


///*******************************************************///

///---------------------------------------------------------////
// Start of Letter D
///---------------------------------------------------------////
```

```
/// Definitions
// Vertical C line (60* of height)
var VerticalD = Height*0.6;
int verticalD= (int) VerticalD;

// Chamfer definitipn (20% of Height; 45 Degrees)
var ChamferD= Height*0.2;
int chamferD= (int) ChamferD;



// Vertical line
c2.Request(Command.MoveRelative, Height);

// Horizontal line
c4.Request(Command.MoveRelative, Height);

// Chamfers
        topic = c2.StartTopic();
c2.Device.Send(Command.MoveRelative, -chamferD, topic.MessageId);
while ( ! topic.IsComplete)
{
        c4.Request(Command.MoveRelative, chamferD);
}

topic.Validate();

// Vertical line
c2.Request(Command.MoveRelative, -verticalD);

// Chamfers
        topic = c2.StartTopic();
c2.Device.Send(Command.MoveRelative, -chamferD, topic.MessageId);
while ( ! topic.IsComplete)
{
        c4.Request(Command.MoveRelative, -chamferD);
}

topic.Validate();

// Horizontal line
c4.Request(Command.MoveRelative, -Height);
```
